# Supplementary material for: PU.1 drives specification of pluripotent stem cell-derived endothelial cells to LSEC-like cells
Source: Cell Death Dis. 2021 Jan 14;12(1):84. doi: 10.1038/s41419-020-03356-2 (PMC7809369; doi:10.1038/s41419-020-03356-2)
Supplement: Supplementary file 1 — Supplementary Figures [file 41419_2020_3356_MOESM1_ESM.pdf]

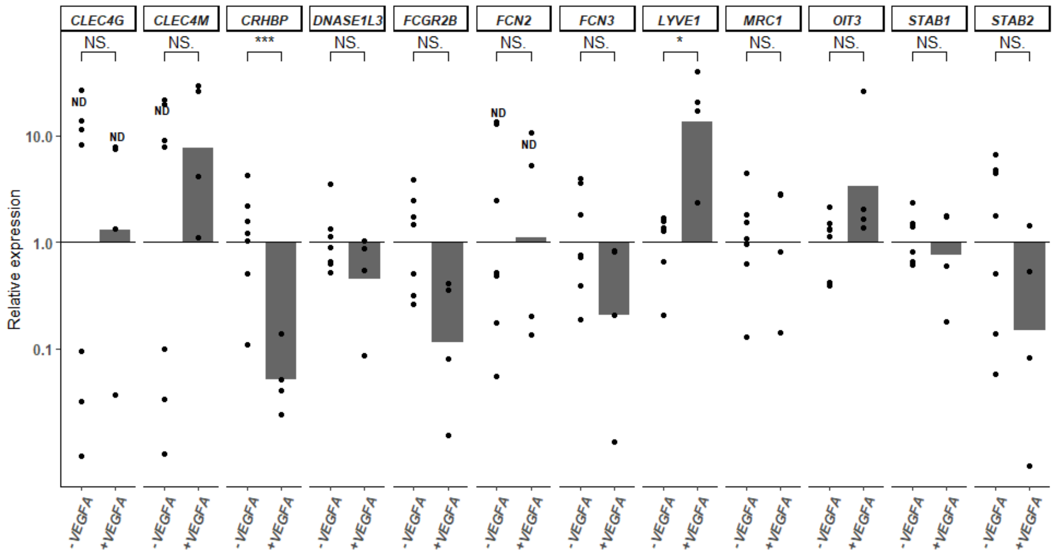

Supplementary Figure 1. Effect of VEGFA addition to the culture media

VEGFA (50ng/ml) was added to the culture media of ETV2-SPI1-ECs, starting from day 6 of differentiation. (N=7 for samples without VEGFA, N=4 for samples with VEGFA) (Student's T-test; * $p<0.05$, ** $p<0.01$, *** $p<0.001$, NS: not significant, ND: not detectable)

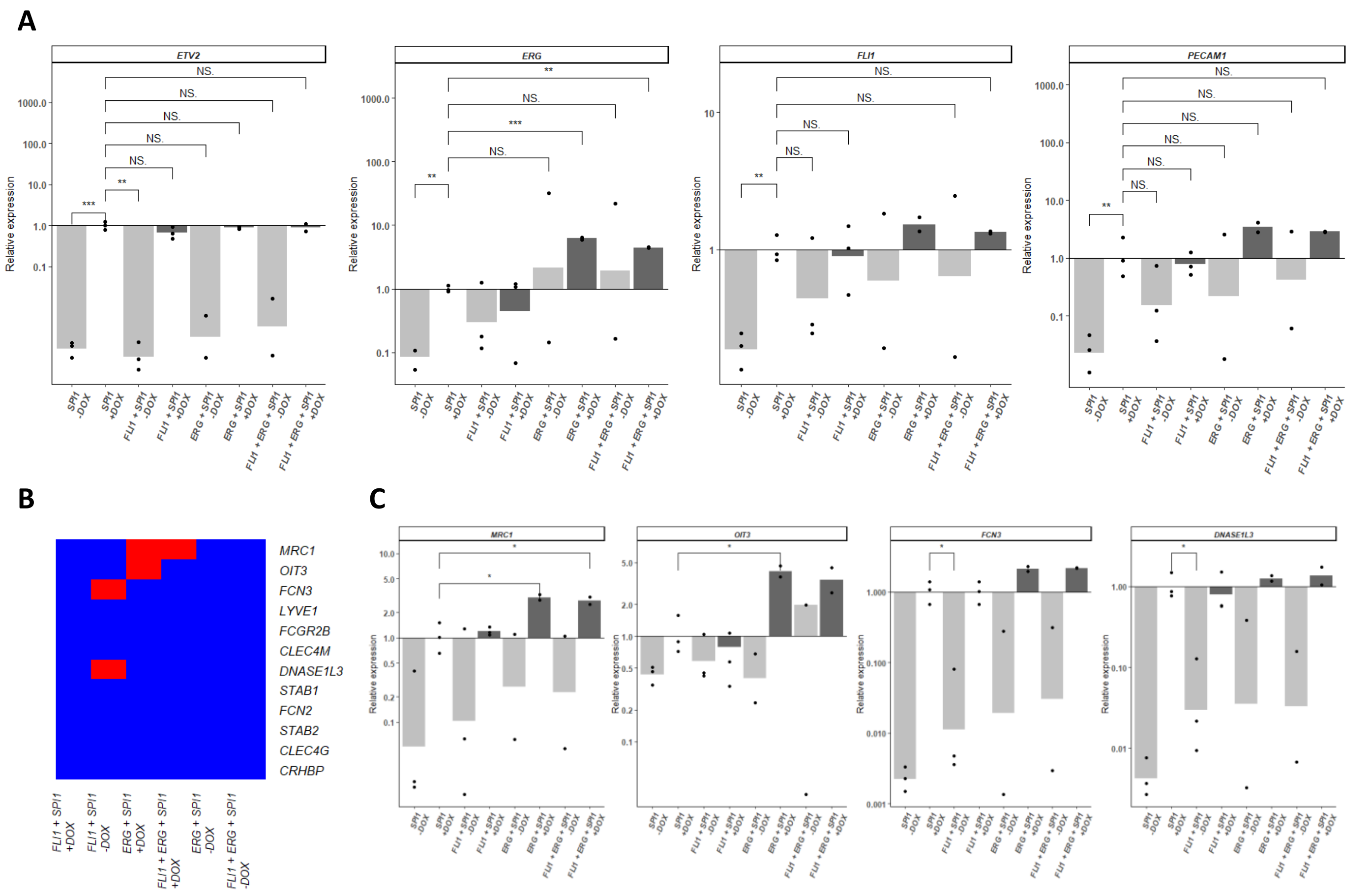

Supplementary Figure 2. Effect of substituting *ETV2* overexpression by lentiviral overexpression of *FLI1* and *ERG*

A. ETV2-ECs were co-transduced with *SPI1*- and *FLI1*- and/or *ERG*- encoding lentiviral vectors, with or without doxycycline-induced *ETV2* overexpression. The expression of endothelial TFs *ETV2*, *ERG*, and *FLI1*, and the endothelial marker *PECAM1* is shown. **B.** Heatmap indicating in red genes that were significantly ($p<0.05$) differentially expressed compared to ETV2-ECs transduced with S*PI1*-encoding lentiviral vector and exposed to doxycycline. **C.** Bar plots of the genes that were significantly affected by substituting *ETV2* overexpression for *FLI1* and/or *ERG* lentiviral overexpression. All statistical significances were assessed with a Student's T-test; * $p<0.05$, ** $p<0.01$, *** $p<0.001$, NS not significant.) (All samples with ERG overexpression: N=2; all other samples: N=3)

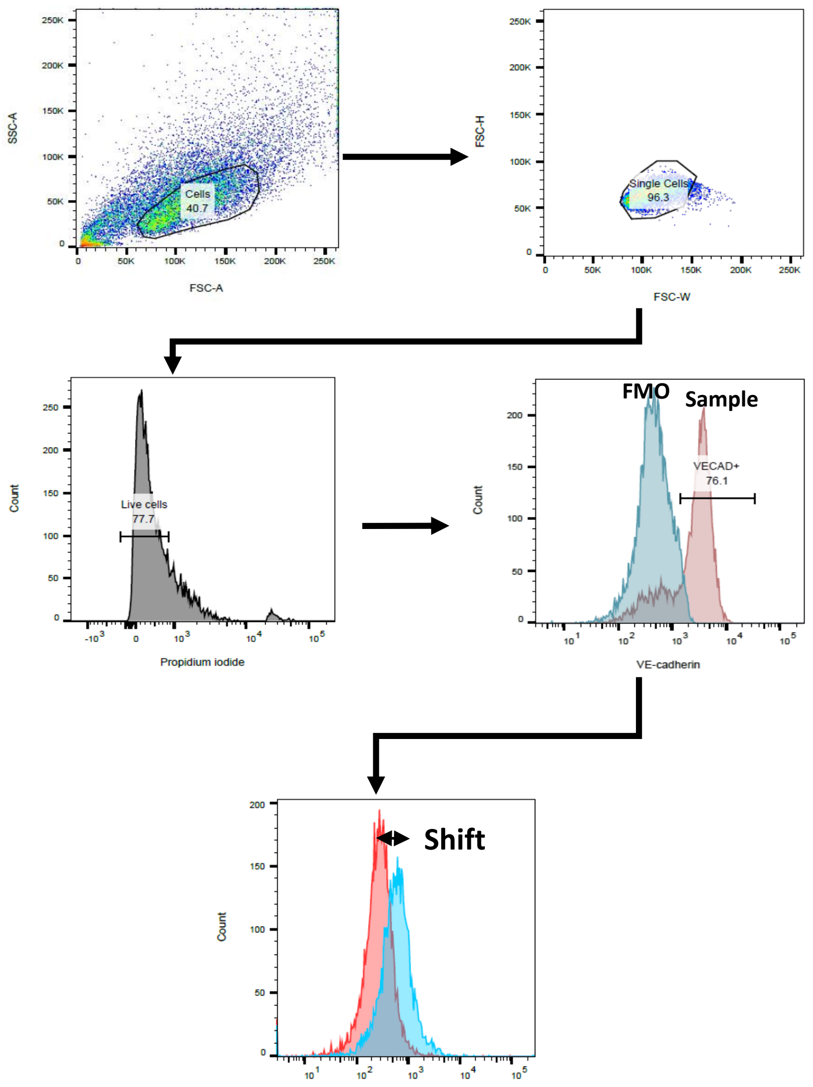

Supplementary Figure 3. Flow cytometry gating strategy

Flow cytometry gating strategy for the measurements of CD32B and MRC1 **(Figure 6E)**. Arrows indicate the order of subsequent gatings. Red and blue density curves in the final plots represent ETV2-ECs (day 12) and ETV2-SPI1-ECs (day 12) respectively. (FMO: Fluorescence Minus One control).

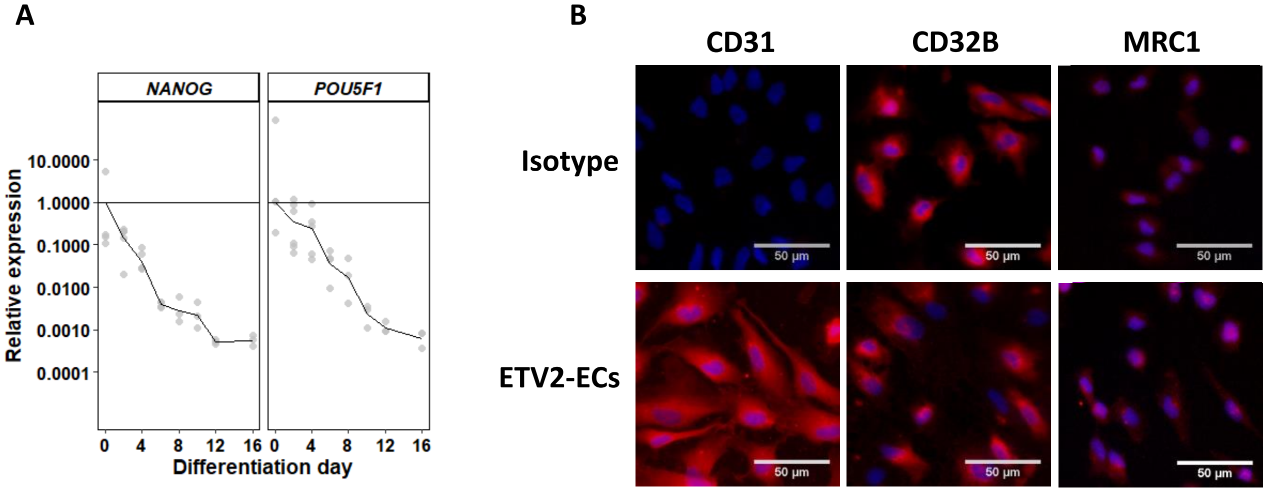

Supplementary Figure 4. Additional characterisation of ETV2-EC endothelial differentiation.
A. Gene expression (RT-qPCR) of pluripotency markers *NANOG* and *POU5F1* throughout a 16-day ETV2-EC endothelial differentiation. **B.** Immunostaining for the endothelial marker CD31, and the LSEC markers CD32B and MRC1. Similar CD32B perinuclear staining was observed in ETV2-ECs compared to the isotype control.

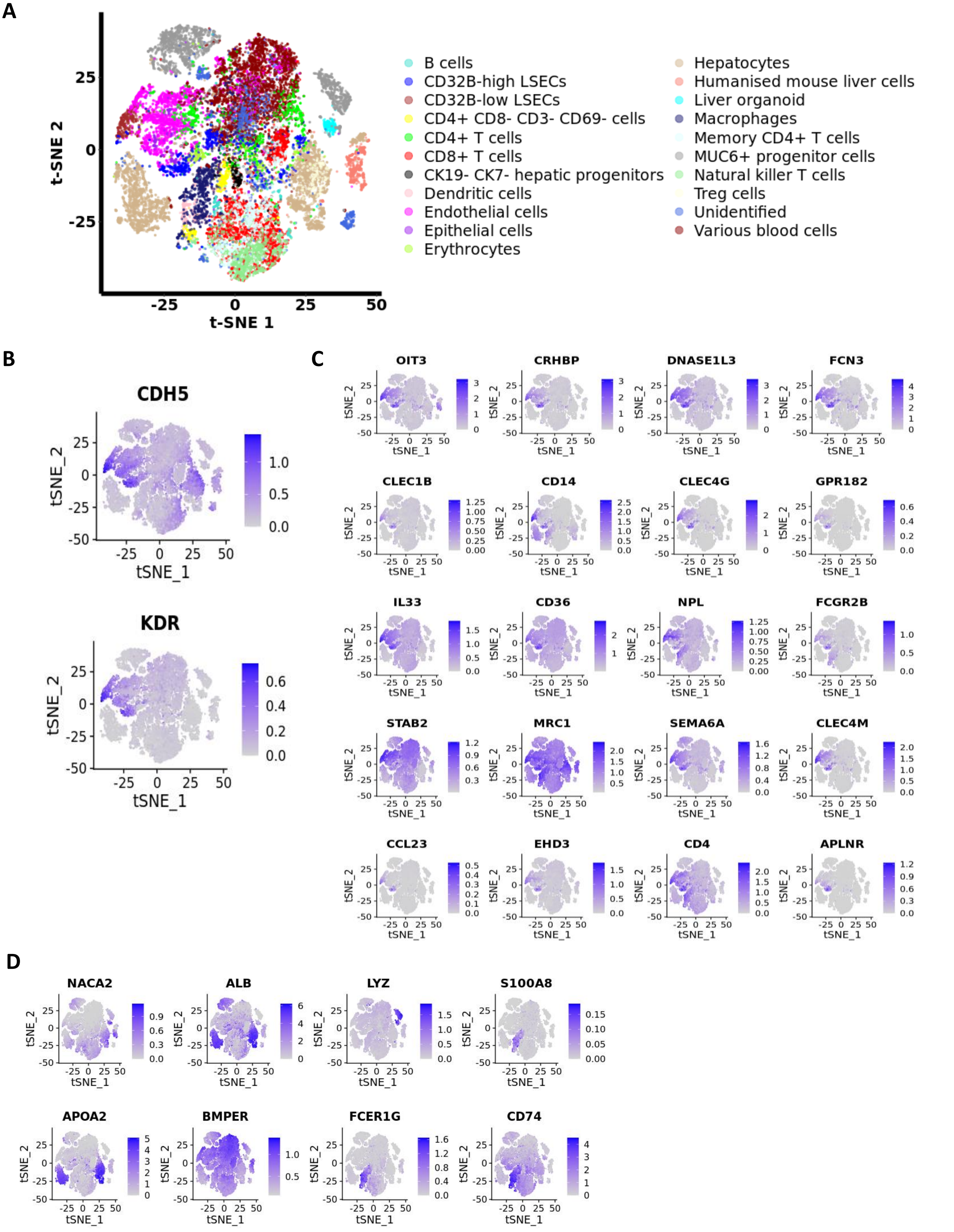

Supplementary Figure 5. Expression of putative LSEC markers in single-cell RNA sequencing data

A. t-SNE representation of the single-cell RNA sequencing data from Aizarani et al. 2019 (42). **B**. Endothelial cells in this dataset were identified by expression of *CDH5* and *KDR*. Genes that were differentially expressed (in the microarray meta-analysis) between LSECs and other endothelial cells were ranked by fold change. Most of the top ranked genes appeared to be specifically expressed in LSECs **(C)**, while some genes ranked high due to contamination with mRNA of other liver cells **(D)**.

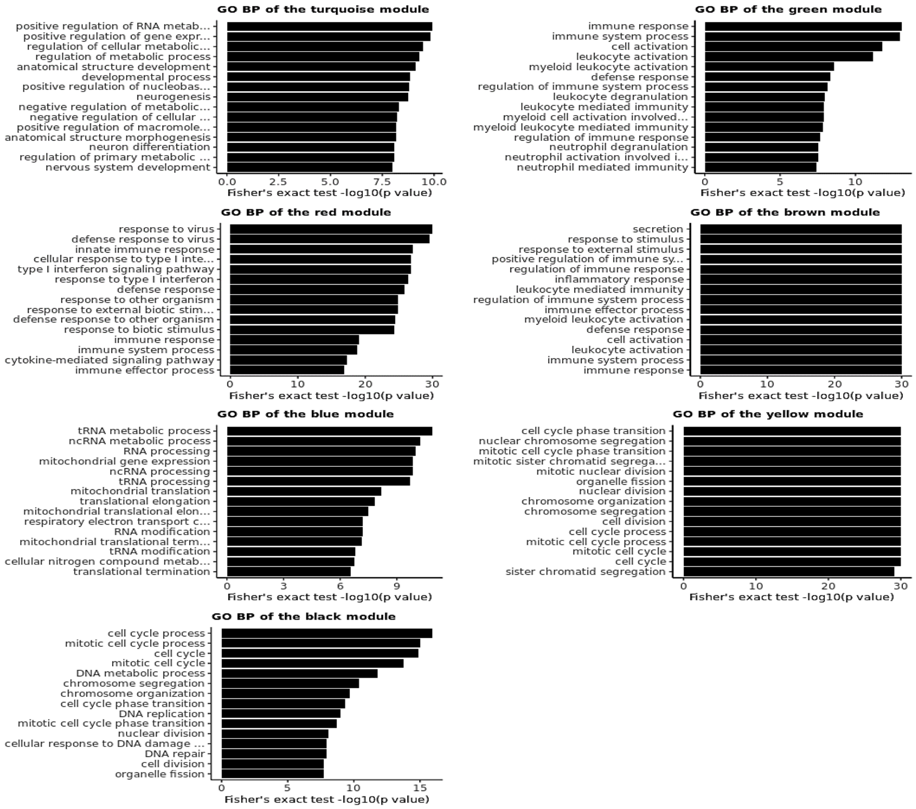

Supplementary Figure 6. Gene ontology enrichment analysis for each of the WGCNA modules

Each of the identified co-expression modules identified by WGCNA was subjected to TopGO Gene Ontology enrichment analysis to identify each module's function. Gene co-expression modules tend to be highly enriched for specific functionality.

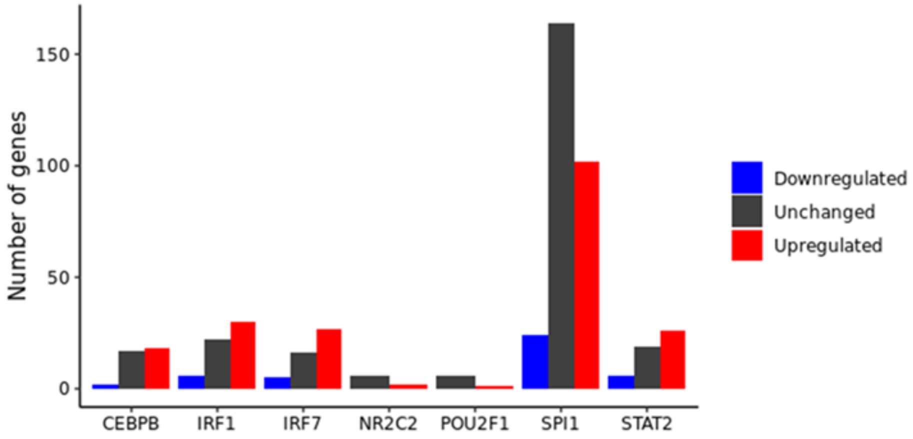

Supplementary Figure 7. Number of genes in the immune response modules directly regulated by each of the candidate transcription factors.

Bars indicate the number of downstream target genes that is downregulated, unchanged, or upregulated in LSECs compared to ETV2-ECs for each of the transcription factors in the three modules related to immune responses.

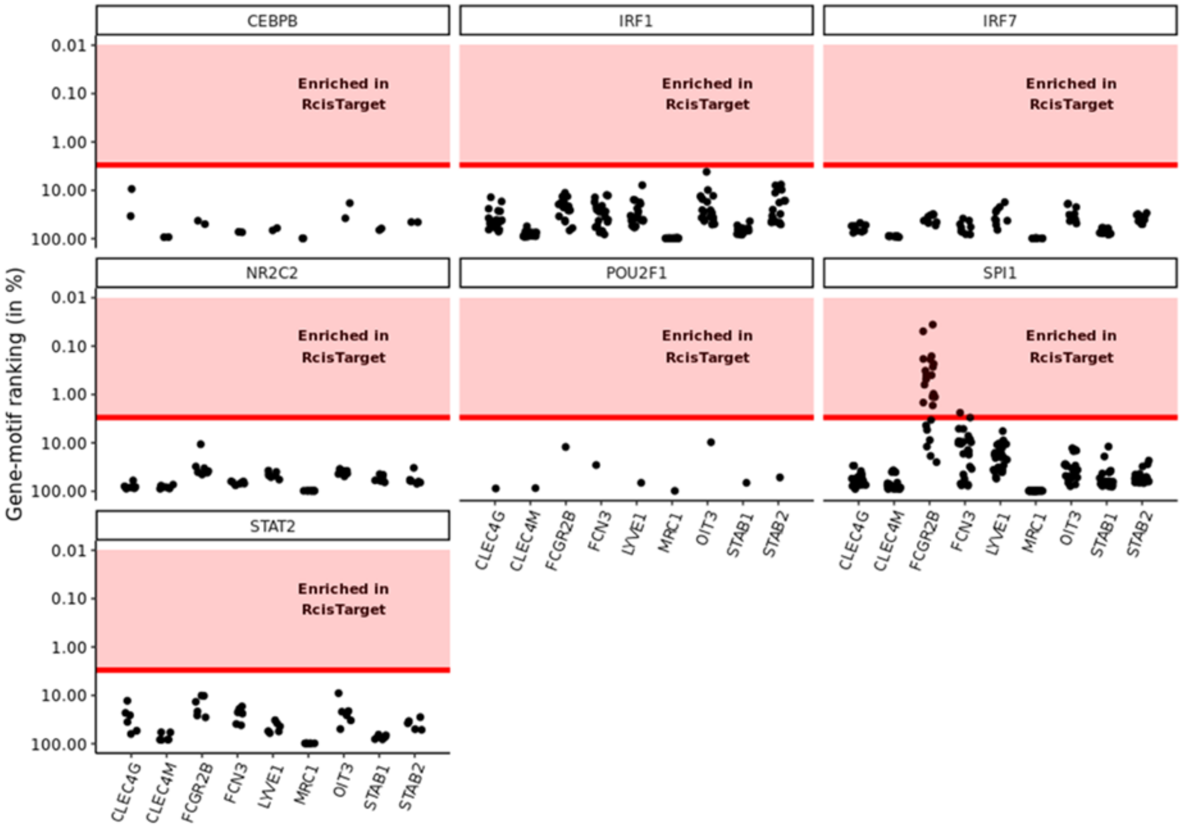

Supplementary Figure 8. Motif ranking for the selected transcription factors and markers.
Each dot represents a motif that is bound by the transcription factor indicated in the respective plot header. Ranks were imported from the RcisTarget gene-motif database (*hg19-tss-centered-10kb-7-species.mc9nr.feather*) for 22,284 RefSeq genes. The cis-regulatory element of a gene with rank 0 for a given motif is mostly likely to be bound by transcription factors that recognise this motif. In the RcisTarget analysis top ranks are converted to top percentages, i.e. the top 3% ranking of each motif contains the 668 genes with the most likely binding on this motif. RcisTarget generally keeps the top 3% (red-shaded area) as a cut-off. Only *FCGR2B* and *FCN3* had likely motif binding targets for PU.1, while none of the other markers were likely to be bound by any of the other transcription factors.

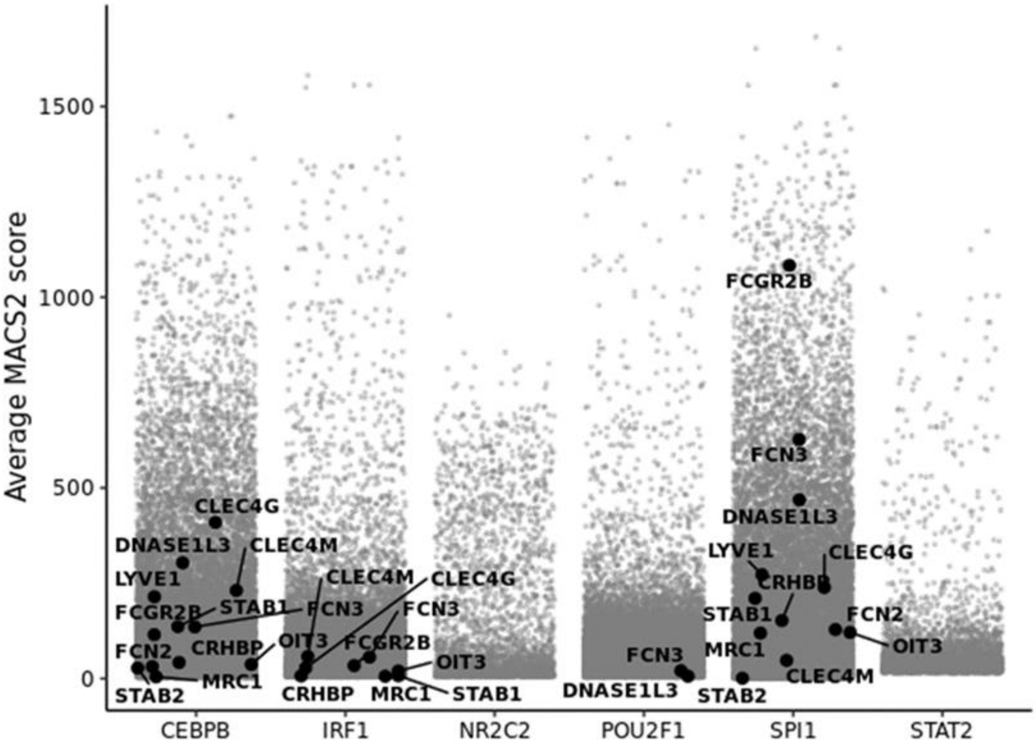

Supplementary Figure 9. *In silico* ChiP-Seq analysis of the immune response transcription factors.

ChiP-Seq of all samples with ChiP for the respective transcription factors were downloaded from the ChiP-Atlas (https://chip-atlas.org/target_genes). Potential target genes were selected within a distance of 10kb before and after the transcription start site (TSS). On the y-axis the MACS2 scores (average of all samples) are plotted. LSEC marker genes are indicated in bold.

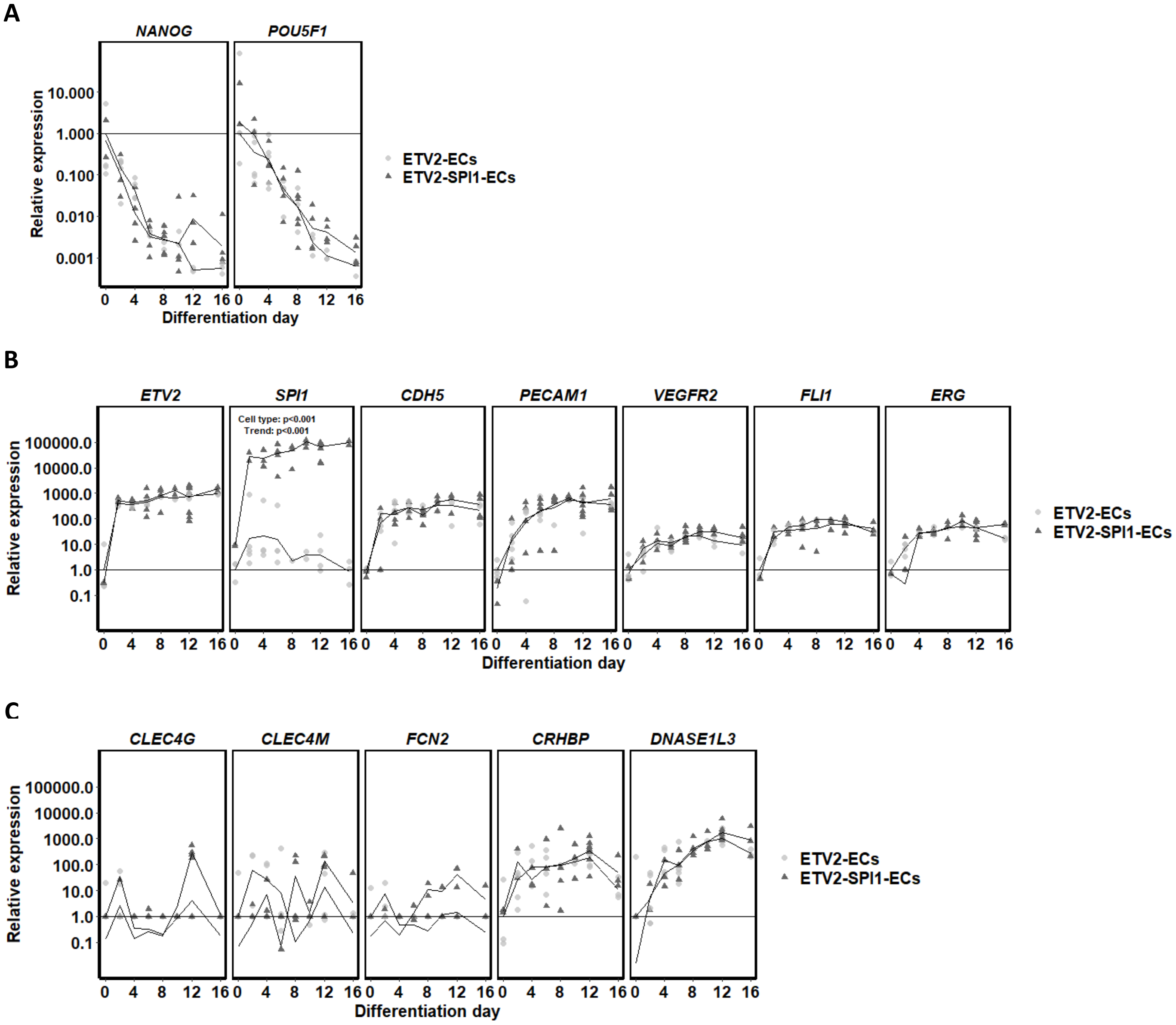

Supplementary Figure 10. Time-course RT-qPCR characterisation of ETV2-ECs and ETV2-SPI1-ECs

A. Expression of pluripotency markers *NANOG* and *POU5F1*. **B.** Expression of overexpressed TFs *ETV2* and *SPI1*, of endothelial-specific genes *CDH5*, *PECAM1*, and *VEGFR2*, as well as endothelial-enriched TFs *FLI1* and *ERG*. **C.** Expression of some of the LSEC markers. All statistical differences were assessed by mixed ANOVA. P-values of significant differences between ETCV2-ECs and ETV2-SPI1-ECs are plotted.

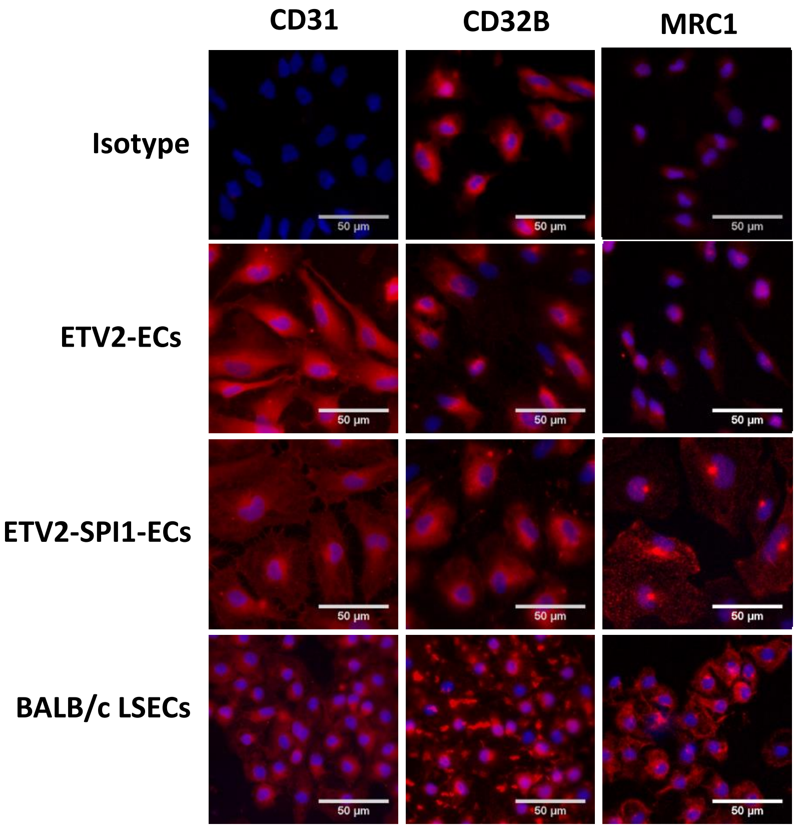

Supplementary Figure 11. Immunostaining characterisation of ETV2-ECs and ETV2-SPI1-ECs

Immunostaining of ETV2-ECs (day 12), ETV2-SPI1-ECs (day 12), and mouse BALB/c LSECs for the endothelial marker CD31, and for the LSEC markers CD32B and MRC1. (Representative images of N=3 biological replicates). Similar CD32B perinuclear staining was observed in ETV2-ECs compared to the isotype control. In ETV2-SPI1-ECs a more homogenous CD32B staining of the entire plasma membrane was observed. (Some images are repeats from Supplementary Figure 4)
